# Six Oligosaccharides’ Variation in Breast Milk: A Study in South China from 0 to 400 Days Postpartum

**DOI:** 10.3390/nu13114017

**Published:** 2021-11-11

**Authors:** Shuang Liu, Xiaokun Cai, Jin Wang, Yingyi Mao, Yan Zou, Fang Tian, Bo Peng, Jiaqiang Hu, Yanrong Zhao, Shuo Wang

**Affiliations:** 1 Tianjin Key Laboratory of Food Science and Health, School of Medicine, Nankai University, Tianjin 300071, China; 2120171314@mail.nankai.edu.cn (S.L.); wangjin@nankai.edu.cn (J.W.); 2120181361@mail.nankai.edu.cn (Y.Z.); pengbo@nankai.edu.cn (B.P.); heric@mail.nankai.edu.cn (J.H.); 2 Abbott Nutrition Research & Development Center, Abbott Ltd., Shanghai 200233, China; xiaokun.cai@abbott.com (X.C.); yingyi.mao@abbott.com (Y.M.); fang.tian@abbott.com (F.T.)

**Keywords:** breast milk, human milk oligosaccharides, lactational stages, variation

## Abstract

This study investigated the variation in oligosaccharide levels in the breast milk of south Chinese mothers in a prolonged breastfeeding period of up to 400 days postpartum. A total of 488 breast milk samples were collected from 335 healthy mothers at five different time points: 0–5 days, 10–15 days, 40–45 days, 200–240 days, and 300–400 days postpartum. A high-performance anion-exchange chromatography-pulsed amperometric detector (HPAEC-PAD) was used to quantify 2′-fucosyllactose (2′-FL), 3-fucosyllactose (3-FL), lacto-N-tetraose (LNT), lacto-N-neotetraose (LNnT), 3′-sialyllactose (3′-SL) and 6′-sialyllactose (6′-SL). In this study, we found six oligosaccharides that were present in breast milk from 0 to 400 days postpartum. The median value ranges of individual oligosaccharide components in this study were 1013–2891 mg/L 2′-FL, 193–1421 mg/L 3-FL, 314–1478 mg/L LNT, 44–255 mg/L LNnT, 111–241 mg/L 3′-SL, and 23–602 mg/L6′-SL. HMO levels decreased over the lactation periods, except for 3-FL, which increased throughout lactation. The predominant fucosylated and sialylated HMOs were 2′-FL and 6′-SL at 40–45 days postpartum and changed to 3-FL and 3′-SL at 200–240 days postpartum. Results from this study showed that lactating women continue to provide their offspring with a high level of 2′-FL one year after delivery, suggesting that 2′-FL may play an important role for infants in early life. Our findings also provide further evidence in support of breastfeeding after one-year postpartum.

## 1. Introduction

Breast milk contains various nutrients to meet infants’ needs for age-appropriate growth in early life [1,2], and the World Health Organization (WHO) recommends exclusively breastfeeding in the first six months and continuing breastfeeding until the age of two or beyond. Several studies have reported that three breastfeeding methods, exclusive breastfeeding, breastfeeding as a primary feeding method, and prolonged breastfeeding, are related to lower risks of asthma, eczema, and obesity later in life [3,4,5,6]. Human milk oligosaccharides (HMOs) are the third most abundant component in breast milk after lactose and lipids [7,8]. Furthermore, as non-digestible carbohydrates [9], HMOs play roles in supporting infant immune development. HMOs are selective substrates for intestinal microbiota, such as Bifidobacterium and Bacteroides [10,11,12], that inhibit the colonization of pathogens by acting as decoys whose structures resemble surface carbohydrates on intestinal epithelial cells to inhibit the binding of pathogens [13,14,15] and support immune maturation by directly interacting with immune cells [16,17,18]. In addition, HMOs contain abundant N-acetylneuraminic acid (sialic acid, Neu5Ac), an important component in brain gangliosides and poly-sialic acid chains contributing to synaptic connections and memory formation [19,20,21].

More than 150 HMOs have been identified in breast milk; they are derived from 5 core monosaccharides, including glucose, galactose, N-acetylglucosamine, fucose, and sialic acid [22,23]. HMOs contain one lactose disaccharide (Galβ1-4Glc) at the reducing end. This lactose subunit is extended by the addition of lacto-N-biose (Galβ1-3GlcNAc) and/or N-acetyllactosamine (Galβ1-4GlcNAc). Such oligosaccharides are referred to as acetylated HMOs with terminal N-acetylglucosamine residues, for example, lacto-N-tetraose (LNT) and lacto-N-neotetraose (LNnT). Furthermore, this lactose subunit and the acetylated HMOs may be further modified by fucose and sialic acid. Oligosaccharides with terminal fucose residues linked by α 1–2, α 1–3, or α 1–4 glycosidic bonds are classified as fucosylated HMOs, such as 2′-fucosyllactose (2′-FL) and 3-fucosyllactose (3-FL). Oligosaccharides with terminal sialic acid residues linked via α 2–3 and α 2–6 glycosidic bonds are classified as sialylated HMOs, such as 3′-sialyllactose (3′-SL) and 6′-sialyllactose (6′-SL). This study focused on six prevalent HMOs: 2′-FL, 3-FL, LNT, LNnT, 3′-SL, and 6′-SL, which are high in breast milk and have been reported to play a role in infants’ health [8,24,25,26].

Lactation and maternal phenotypic secretor types play important roles in HMO concentration and profile. The concentration of total HMOs in colostrum is higher than that in the later stages of lactation, ranging from 20–25 g/L in colostrum but decreasing to 5–15 g/L in mature milk [7,8]. Maternal phenotypic secretor types are classified as ‘secretor’ and ‘non-secretor’ depending on the expression of the Secretor (Se) gene encoding α 1-2-fucosyltransferase (FUT2). The maternal phenotypic secretor type plays a role in the level of fucosylated HMOs [26]. The milk of secretor mothers with FUT2 expression is rich in α 1–2 linked fucosyl-oligosaccharides, such as 2′-FL and Lacto-N-fucopentaose I (LNFP I). By contrast, milk from non-secretor mothers with a negative Se locus does not contain a large number of α 1–2-linked fucosyl-oligosaccharides but contains many α 1–3-linked fucosyl-oligosaccharides, such as 3-FL and Lacto-N-fucopentaose III (LNFP III) [27,28,29]. The ratio of secretor and non-secretor phenotypes in the literature is 64–89%:11–36% [26,27,30,31,32,33,34].

Although a growing number of studies have reported HMO concentration and variation [25,26,27,34,35,36], few studies on HMO concentration and variation over a one-year postpartum period have been conducted. In this study, we report the HMO levels in 448 breast milk samples among 335 healthy women who participated in a study from 0 to 400 days postpartum. Moreover, this is the first study to report the HMO levels in milk one year postpartum from south Chinese mothers. This work was part of the Maternal Nutrition and Infant Investigation (MUAI) study designed to investigate (1) the composition and functionality of breast milk nutrients, (2) factors affecting nutrient levels in breast milk, and (3) the relationship between nutrients and infant health. Human milk oligosaccharides are associated with various benefits. Investigating HMO levels over a prolonged breastfeeding period contributes to a better understanding of HMO-associated health benefits.

## 2. Materials and Methods

### 2.1. Study Design and Participants

In this study, 335 healthy women were recruited in Clifford Hospital, Panyu District, Guangzhou City, Guangdong Province, China, between March 2018 and June 2019. Breast milk samples were collected at five different time points: 0–5 days, 10–15 days, 40–45 days, 200–240 days, and 300–400 days post-partum. This study also collected maternal and infant information and dietary questionnaires at each sampling time, which were conducted by hospital doctors and trained students. The inclusion criteria were that the pregnant women had lived in the area for more than two years, were aged 20–35 years, planned to breastfeed for more than 3 months, had singleton pregnancies, and had a gestational age of 37–42 weeks. Subjects were excluded if the mother–infant pair had any health conditions, including chronic diseases and acute or chronic infectious diseases, or were taking drugs during pregnancy or lactation that could affect nutrient metabolism. This study was conducted according to the guidelines laid out in the Declaration of Helsinki, and all procedures involving human subjects were approved by the Ethics Committee of Clifford Hospital and registered in the China Clinical Trial Center (ChiCTR1800015387) as a part of the Maternal Nutrition and Infant Investigation (MUAI) study. Written informed consent was obtained from all participants.

### 2.2. Sample Collection and Preservation

A standardized sampling procedure was applied for all mothers. All mothers were asked to empty the milk from one breast with a manual or an electric breast pump between 8:00 and 11:00 a.m. on the collection day. The breast milk was mixed thoroughly, the collection tube was inverted 6 times, and 30–50 mL was poured into a sterile light-proof centrifuge tube, except during 0–5 days and 10–15 days postpartum, when we collected 8–10 and 10–30 mL, respectively. The remaining milk was returned to the mother for feeding to the infant. All breast milk samples were transported to Abbott Nutrition Research and Development Centre in Shanghai and stored for later analysis. All the milk samples were stored at a constant temperature (−80℃) during transportation and storage.

### 2.3. HMO Analysis×

A high-performance anion-exchange chromatography with pulsed amperometric detector (HPAEC-PAD) was used to measure HMOs in breast milk as previously described with some modifications [37]. Briefly, a 200 μL homogenized milk sample was diluted 10 times with Milli-Q water (milk samples from 0–5 days postpartum was diluted 20 times for quantification) and then vortexed for 1 min to mix thoroughly. The mixture was filtered with a 0.22 μm nylon filter to remove proteins and lipids. The filtrate was transferred to a vial. The identification of HMOs was performed on a Thermo Fisher HPAEC ICS 5000 series (Thermo, Waltham, MA, USA) system equipped with a separation column (CarboPacTM PA1, 4 × 150 mm, Thermo) connected with a guard column (CarboPacTM PA1, 4 × 50 mm, Thermo). The column and detector temperature were maintained at 25 °C. PAD (Ag) and carbohydrate tetrad potential were used during the analysis. The injection column was 10 mL. The oligosaccharides were separated with a gradient of water (Eluent A), sodium hydroxide (500 mmol/L, Eluent B), and sodium acetate solution (300 mmol/L, Eluent C) at 1.0 mL/min. The gradient used for the analysis of 2′-FL, 3-FL, LNT, and LNnT was as follows: 0.00–20.00 min, 84% A, 16% B, and 0% C; 20.01–30.00 min, 75% A, 25% B, and 0% C; 30.01–43.00 min, 67% A, 25% B, and 8% C; 43.01–48.00 min, 0% A, 20% B, and 80% C; 48.01–63.00 min, 84% A, 16% B, and 0% C. The gradient used for the analysis of 3′-SL and 6′-SL was as follows: 0.00–19.00 min, 60% A, 20% B, and 20% C; 19.01–23.00 min, 0% A, 20% B, and 80% C; 23.01–30.00 min, 60% A, 20% B, and 20% C. The quantification of the six HMOs was based on individual standard response curves. The standards for 3-FL (>95%), LNT (>90%), and 3′-SL (>98.5%) were purchased from Carbosynth (Berkshire, UK). The standards for 2′-FL, LNnT, and 6′-SL were provided by Abbott (Chicago, IL, USA). The software, Chromeleon 6.8 and Chromeleon 7.2 (Thermo, Waltham, MA, USA), was used to control the process and compute the retention time and peak areas with the standards.

### 2.4. Method Validation

The method’s linearity was within the given quantitation range, and the correlation coefficients were all greater than 0.999. The limit of quantitation (LOQ) was determined by adding a given amount of HMO, and the limit of detection (LOD) level was calculated with three times the signal-to-noise (S/N) ratio. The LODs were as follows: 0.4 mg/L2′FL, 5.2 mg/L 3-FL, 2.5 mg/L LNT, 2.8 mg/L LNnT, 1.8 mg/L 3′-SL, and 1.0 mg/L 6′-SL. The LOQs were 9.8 mg/L 2′FL, 19.8 mg/L 3-FL, 19.7 mg/L LNT, 19.9 mg/L LNnT, 9.9 mg/L 3′-SL, and 9.9 mg/L 6′-SL. The precision and accuracy were determined by 6 replication tests of one milk sample and the spiked experiments were repeated 6 times at 50% and 100% of each HMO concentration in the same milk sample. The relative standard deviation (RSD) was below 3.7%, and the recoveries of each HMO were all within 91.8–106.1% with good precision (RSD% < 2.1%). In 488 milk samples, the proportions of >LOD were as follows: 2′-FL: 99%, 3-FL: 100%, LNT: 100%, LNnT: 99%, 3′-SL: 100%, and 6′-SL: 100%; the proportions of >LOQ were as follows: 2′-FL: 97%, 3-FL: 100%, LNT: 100%, LNnT: 89%, 3′-SL: 100%, and 6′-SL: 93%.

### 2.5. Statistical Analysis

All exploratory and descriptive statistical analyses were performed with SPSS 25.0. HMO concentrations were reported as medians (P25, P75) since the data presented a non-normal distribution. The minimum sample size was calculated by the formula ((z∗SD/E)^2^). E was the average value of the HMO concentration multiplied by the allowable error of the method. The SD was the standard deviation of the HMO concentrations, and z = 1.96. The variation of the HMO concentrations over five lactational stages was explored with independent nonparametric tests (Kruskal–Wallis one-way ANOVA, pairwise). According to the distribution of the 2′-FL level, the milk samples were separated into two groups: low and high 2′-FL level groups. The HMO levels in the two groups were compared by a two-independent sample nonparametric test (Kolmogorov–Smirnov Z). The nonparametric Spearman test was performed to investigate the correlation between individual HMOs. All statistical analyses were considered significant at *p* < 0.05 (two-sided). The sum of the concentrations of 2′-FL, 3-FL, LNT, LNnT, 3′-SL, and 6′-SL represented the total HMO concentrations.

## 3. Results

### 3.1. Basic Characteristics

The characteristics of the participating mothers at each sampling time are listed in Table 1. A total of 488 milk samples were collected from 335 healthy lactating mothers: 96 at 0–5 days, 96 at 10–15 days, 104 at 40–45 days, 100 at 200–240 days, and 92 at 300–400 days postpartum. Overall, 46 mothers had samples taken at the first three time points. The average (SD) age of the participant mothers was 29.6 (3.6) years. The gestational age of the participant mothers was 39.2 (1.4) weeks. The pre-pregnant BMI and pre-delivery BMI of the participating mothers were 20.2 (2.7) kg/m^2^ and 25.5 (3.2) kg/m^2^, respectively. The weight gain during pregnancy was 13.5 (4.9) kg. The rate of vaginal delivery was 76%.

### 3.2. HMO Levels over Lactational Stages

The changes in HMO levels from 0 to 400 days postpartum are shown in Table 2. 2′-FL levels decreased while 3-FL levels increased from 0 to 240 days and then remained stable. LNT and 6′-SL levels increased from colostrum to transitional milk and decreased significantly in mature milk. By contrast, the 3′-SL levels showed a ‘U’ curve from 0 to 400 days. LNnT level continuously declined throughout the lactational stages. The 6′-SL level was higher than the 3′-SL level at 0–45 days postpartum, while the level of 3′-SL was higher than that of 6′-SL at 200–400 days postpartum. The level of LNT was higher than that of LNnT throughout all sampling periods. Among the six HMOs, the 2′-FL level was the highest among all the HMOs at 0–45 days postpartum, and subsequently, the level of 3-FL became the highest of all the HMOs at 200–400 days postpartum. The total HMO level decreased throughout the lactational stages but remained at 59% of the total level observed at 0–5 days postpartum.

### 3.3. HMO Levels in High and Low 2′-FL Level Groups

**Figure 1:**
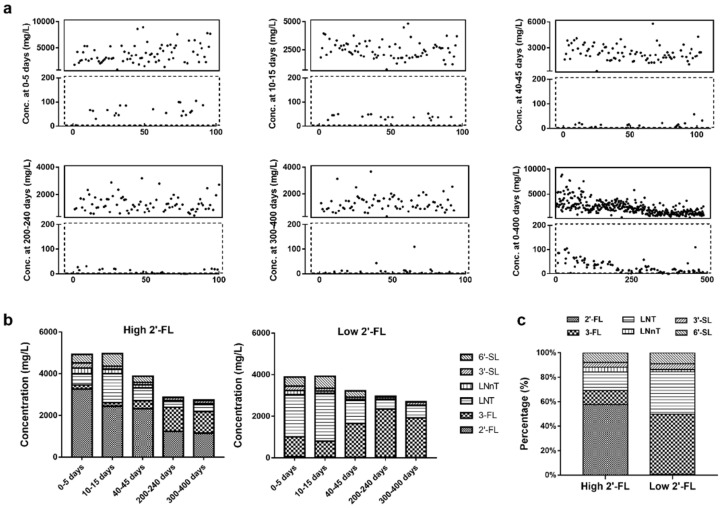
The distribution of 2′-FL concentration, the differences in HMO concentrations, and percentages between high and low 2′-FL level groups. (**a**) The distribution of 2′-FL concentration at 0–5 days, 10–15 days, 40–45 days, 200–240 days, 300–400 days, and 0–400 days postpartum. The ordinate is 2′-FL concentration, and the abscissa is the number of mothers. The solid line represents the high 2′-FL level group; the dotted line represents the low 2′-FL level group. (**b**) The concentration of six HMOs in high and low 2′-FL level groups from 0 to 400 days postpartum. (**c**) The percentage of six HMOs in high and low 2′-FL level groups from 0 to 400 days postpartum. Conc., concentration; 2′-FL, 2′-fucosyllactose; 3-FL, 3-fucosyllactose; LNT, lacto-N-tetraose; LNnT, lacto-N-neotetraose; 3′-SL, 3′-sialyllactose; 6′-SL, 6′-sialyllactose; High 2′-FL, high 2′-FL level group; Low 2′-FL, low 2′-FL level group.

2′-FL was detected in 484 (99.2%) out of 488 breast milk samples. Based on the 2′-FL level of 200 mg/L, breast milk samples were divided into the high 2′-FL group, which accounted for 79% of the total population, and low 2′-FL level groups, which accounted for 21% of the total number of samples (Figure 1a). The median level of 2′-FL was 2001 mg/L in the high 2′-FL group and 20 mg/L in the low 2′-FL group from 0 to 400 days.

The breast milk samples in the high 2′-FL group also contained a higher level of LNnT and lower levels of 3-FL and LNT than the low 2′-FL level group (Figure 1b). Notably, 2′-FL was the most abundant HMO of the six HMOs in the high-2′-FL group from 0 to 400 days postpartum. By contrast, 3-FL was the most abundant HMO in the low 2′-FL level group (Figure 1c).

The concentration and variation of the individual HMOs and the total HMOs from 0 to 400 days postpartum are shown in Table 3. The total level of six HMOs in the high 2′-FL group was 1.2 times higher than that in the low 2′-FL group from 0 to 400 days postpartum (*p* < 0.01). The levels of 2′-FL, 3-FL, LNT, and LNnT were significantly different between the high and low 2′-FL level groups (*p* < 0.01).

### 3.4. Correlations between Individual HMOs

There was a range of correlations between different HMOs. 2′-FL was significantly correlated with 3-FL (r = −0.77; *p* < 0.01), LNnT (r = 0.64; *p* < 0.01), 3′-SL (r = 0.24; *p* < 0.01), and 6′-SL (r = 0.40; *p* < 0.01) from 0 to 400 days postpartum. 3-FL was inversely correlated with 2′-FL (r = −0.77; *p* < 0.01), LNT (r = −0.39; *p* < 0.01), LNnT (r = −0.72; *p* < 0.01), 3′-SL (r = −0.20; *p* < 0.01), and 6′-SL (r = −0.59; *p* < 0.01) from 0 to 400 days postpartum. LNT was directly correlated with LNnT (r = 0.37; *p* < 0.01) and 6′-SL (r = 0.67; *p* < 0.01) from 0 to 400 days postpartum. LNnT was correlated with 3′-SL (r = 0.31; *p* < 0.01) and 6′-SL (r = 0.60; *p* < 0.01) from 0 to 400 days postpartum. 3′-SL was directly correlated with 6′-SL (r = 0.24; *p* < 0.01) from 0 to 400 days postpartum.

## 4. Discussion

This study is the first to report HMO levels in Chinese breast milk over a prolonged breastfeeding period up to 400 days postpartum and use 2′-FL levels to separate breast milk samples into high and low 2′-FL level groups. Our study investigated the variation in HMOs and reported the range of HMO levels in Chinese women’s breast milk from 0 to 400 days postpartum. In summary, HMO levels decreased, except for 3-FL, which is consistent with existing reports [26,35]. Furthermore, HMO profiles changed in later lactational stages. Compared with the HMO ranges in the milk of Chinese mothers in other studies (Table 4), the HMO ranges in this study were larger than the ranges reported by Austin et al. [35], which measured 10 different HMO levels in breast milk from 5 to 240 days postpartum in Beijing, Suzhou, and Guangzhou. Notably, in the current study, the HMO levels from 0 to 5 days and 300 to 400 days postpartum were included. Analytical variation, such as the sample preparation, testing method, and social characteristics of the donors, including geographical distribution, may contribute to moderate differences between study results [24,26,27,29,34,36,38,39].

In this study, 2′-FL was present in breast milk throughout the sampling period at a relatively high level (2 g/L from 0 to 45 days and 1 g/L from 200 to 400 days postpartum). Maternal breast milk continues to provide the offspring with a higher level of 2′-FL for one year after delivery, suggesting that 2′-FL might be important for infants in early life. 2′-FL has attracted more attention in recent years. Several studies have documented the beneficial effects of 2′-FL in shaping the intestinal microbiota, inhibiting pathogen adhesion, and attenuating inflammation [40,41,42,43]. In addition, clinical studies have shown that the addition of 2′-FL to infant formula promotes health benefits for infants. For example, 2′-FL-fortified infant formula supports the developing immune system of infants more similarly to breast milk [44,45,46]. In recent years, studies have divided breast milk phenotypes into secretor and non-secretor groups on the basis of the presence of 2′-FL [31,33]. Typically, non-secretor milk does not contain 2′-FL. However, in this study, 2′-FL was present in breast milk throughout the lactation period and was detected in 99.2% of the samples subjected to the methods described herein. A clear separation was found in the distribution of 2′-FL levels such that the breast milk samples were classified into high and low 2′-FL level groups as opposed to 2′-FL secretor and non-secretor groups. Seventy-nine percent of breast milk samples were assigned to the high 2′-FL level group, and 21% were assigned to the low 2′-FL level group. This observation was close to the secretor group and non-secretor group ratio in Germany based on the Lewis blood type [26]. Our results suggest that breast milk from non-secretor mothers also contains low levels of 2′-FL. Conceivably, 2′-FL is present at some level in most breast milk. This study also provided the range of 2′-FL concentrations in the high and the low 2′-FL groups, which could help future investigators understand the health benefits of different breast milk phenotypes.

With a prolonged sampling time, we found that 3-FL levels exceeded 2′-FL levels in the late lactation period and became the predominant HMO compared with other HMOs. As opposed to 2′-FL, 3-FL levels increased throughout the lactational stages by about 4.1 times. In addition, 3-FL was the most abundant HMO of the six HMOs in the low 2′-FL level group at each sampling point. Available evidence indicates that the function of 2′-FL and 3-FL is slightly different, although both terminate in a fucose monosaccharide. In vitro fermentation experiments conducted by Ruiz-Moyano et al. [12] demonstrated that multiple *Bifidobacterium* subspecies use 3-FL, but only certain *Bifidobacterium* subspecies could utilize 2′-FL. In a study conducted by Bienenstock et al. [47], 2′-FL and 3-FL decreased contractility in the murine colon, and the effect of 3-FL was twice that of 2′-FL. Additionally, 3-FL showed a stronger inhibition of the adhesion of enteropathogenic *Escherichia coli* to the intestinal human cell line Caco-2 than 2′-FL [48]. These findings suggest that 3-FL plays a role in the later stage of infancy.

We also observed that the predominant sialylated HMO changed from 6′-SL to 3′-SL between the periods of 40–45 days and 200–240 days postpartum. The 6′-SL level decreased throughout the lactational stages, while the 3′-SL level was relatively stable throughout the lactation period. 6′-SL was high in breast milk at the beginning of lactation, and its level at 10–15 days postpartum was 26 times higher than that at 300–400 days postpartum. 6′-SL may play an important role in brain development in early life since the brain contains a large amount of sialic acid in gangliosides and polysialic acid chains [49]. In the early stages of life, the synthesis pathway of sialic acid is restricted [50]. Animal experiments in rats have shown that sialylated oligosaccharides improve learning ability and increase brain ganglioside levels [51]. Therefore, sialylated HMOs may provide building blocks that support brain development. In fact, recent clinical research showed that breast milk levels of 6′-SL (one month postpartum) are positively associated with neurodevelopment in breast-fed infants by 18 months of age [52]. Moreover, 73% of sialic acid in breast milk is associated with oligosaccharides [19], which may be readily absorbed and utilized in the gut of infants. This evidence shows that sialylated HMOs in breast milk may be an important way to obtain sialic acid for infants in early life and play an important role in brain development and cognition.

In the high and the low 2′-FL level groups, we found that the profiles of HMOs were significantly different. Compared with the low 2′-FL level group, breast milk samples from the high 2′-FL level group contained higher LNnT levels and lower 3-FL and LNT levels. This observation aligns with those of previous studies in that the amount of fucosylated and acetylated HMOs varied among the milk of secretor types [26,29,53]. Current research indicates the Secretor (Se) gene encoding α 1–2-fucosyltransferase (FUT2) synthesizes α 1–2-linked fucosylated HMOs, and the Lewis (Le) gene encoding α 1–3-fucosyltransferase (FUT3) synthesizes 1–3- or α 1–4-linked fucosylated HMOs [26,29,30]. Conceivably, the high and low 2′-FL level groups have different FUT2 and FUT3 related enzyme expression and activities; in addition, other Se- and Le-independent FUTs may also impact the composition of HMOs [7]. We also found that LNT is the most abundant acetylated HMO in the high and the low 2′-FL level groups, revealing that LNT is a prevalent HMO present in breast milk regardless of the secretor type of the nursing mother. LNT level was higher than LNnT throughout all sampling periods, suggesting an important role for this prevalent acetylated HMO.

The results of this study support the WHO’s recommendation to continue breastfeeding after the first six months of life by providing evidence that HMO levels in breast milk at 300–400 days postpartum account for 59% of the total levels in colostrum. Complementary foods added to an infant’s diet from six months of age cannot replace the benefits of HMOs in breast milk, as there are no food sources containing oligosaccharides with the same diversity and abundance as HMOs in breast milk, although the structure of some oligosaccharides in the milk of non-human mammals is similar to HMOs [54]. For example, the levels of bovine milk oligosaccharides (BMOs) are about 100 times lower than those of the HMOs in breast milk and contain few or no fucosylated oligosaccharides, and the proportion of sialylated oligosaccharides is about 70%, which is higher than the proportion of HMOs (12–14%) found in breast milk [55].

In this study, we discovered that the concentrations of the predominant fucosylated, acetylated, and sialylated HMOs changed from 40 to 45 days postpartum to 200 to 240 days postpartum. However, this study could not determine the specific time of this change because of the large time interval from 45 days to 200 days. Future studies could be conducted to narrow down the time interval. It was not possible to calculate the infants’ daily intake of HMOs since the 24 h milk volume intake of the infants was not recorded in this study. Note that compared with other studies, the sampling time (from 8 to 11 am) and population (healthy mother–infant pairs) in this study must be considered. In addition, the relationship between the Se and Le genes in mothers and the HMO levels in Chinese breast milk need to be further studied.

## 5. Conclusions

This study reported the variation of six HMO levels in the breast milk of south Chinese mothers up to 400 days postpartum. Over the course of lactation, most HMO levels decreased, except for 3-FL. Maternal milk continues to provide the offspring with a higher level of 2′-FL for one year after delivery, suggesting that 2′-FL may be beneficial for infants in early life. Additionally, changes in the predominant fucosylated, acetylated, and sialylated HMOs in breast milk may indicate that HMO requirements differ as infants grow and that HMOs act synergistically to regulate infants’ nutrition. Our results provide a deeper understanding of HMO levels in Chinese mothers in a prolonged breastfeeding period, supporting continual breastfeeding after one year postpartum, and may help to further develop HMO-fortified infant formulas.

## Figures and Tables

**Table 1:** Characteristics of participant mothers at each sampling time (means ± SD or *n* (%)).

Characteristic	Mothers (*n*)				
	0–5 Days (*n* = 96)	10–15 Days (*n* = 96)	40–45 Days (*n* = 104)	200–240 Days (*n* = 100)	300–400 Days (*n* = 92)
Age (years)	28.7 ± 3.1	28.4 ± 3.1	28.6 ± 3.0	30 ± 4	30 ± 4
Gestational age (weeks)	39.4 ± 1.1	39.4 ± 1.2	39.4 ± 1.1	39 ± 1	39 ± 2
Pre-pregnancy BMI (kg/m^2^)	20.3 ± 3.6	20.4 ± 3.5	20.3 ± 3.3	20.1 ± 2.2	20.2 ± 2.4
Pre-delivery BMI (kg/m^2^)	25.8 ± 4.0	25.9 ± 3.7	25.8 ± 3.7	25.1 ± 2.8	25.3 ± 2.9
Gestational weight gain (kg)	14.2 ± 4.6	14.3 ± 4.3	14.1 ± 4.8	12.8 ± 4.9	12.9 ± 5.0
Vaginal delivery	74 (77%)	76 (79%)	84 (81%)	77 (77%)	69 (75%)
Primipara	58 (60%)	66 (68%)	74 (71%)	58 (58%)	63 (67%)

**Table 2:** Concentration of human milk oligosaccharides (HMOs) from 0 to 400 days postpartum (median (p25, p75)) (mg/L).

HMOs	Days Postpartum				
	0–5 Days (*n* = 96)	10–15 Days (*n* = 96)	40–45 Days (*n* = 104)	200–240 Days (*n* = 100)	300–400 Days (*n* = 92)
2′-FL	2891 ^a^ (1715, 4343)	2160 ^a,b^ (1672, 2816)	2063 ^b^ (1376, 2685)	1033 ^c^ (600, 1520)	1013 ^c^ (598, 1478)
3-FL	272 ^c^ (155, 561)	193 ^c^ (128, 381)	480 ^b^ (330, 801)	1421 ^a^ (887, 1921)	1128 ^a^ (826, 1477)
LNT	744 ^b^ (373, 1442)	1478 ^a^ (1077, 2038)	748 ^b^ (487, 1025)	314 ^c^ (204, 465)	361 ^c^ (241, 527)
LNnT	255 ^a^ (188, 404)	183 ^b^ (126, 260)	117 ^c^ (69, 182)	44 ^d^ (23, 81)	44 ^d^ (19, 75)
3′-SL	241 ^a^ (201, 308)	141 ^b^ (124, 162)	111 ^c^ (96, 133)	117 ^c^ (97, 135)	136 ^b^ (114, 162)
6′-SL	409 ^b^ (307, 517)	602 ^a^ (522, 770)	300 ^c^ (218, 370)	39 ^d^ (24, 55)	23 ^d^ (15, 40)
Total HMOs	5120 ^a^ (4224, 6326)	4995 ^a^ (4397, 5417)	3913 ^b^ (3482, 4475)	3084 ^c^ (2752, 3377)	2891 ^c^ (2649, 3157)

2′-FL, 2′-fucosyllactose; 3-FL, 3-fucosyllactose; LNT, lacto-N-tetraose; LNnT, lacto-N-neotetraose; 3′-SL, 3′-sialyllactose; 6′-SL, 6′-sialyllactose. Total HMO concentration was calculated as the sum of 2′-FL, 3-FL, LNT, LNnT, 3′-SL, and 6′-SL. ^a,b,c,d^ values within a row in individual HMO with unlike superscript letters were significantly different (*p* < 0.05) according to the independent nonparametric test (Kruskal–Wallis one-way ANOVA, all pairwise).

**Table 3:** Concentrations of human milk oligosaccharides (HMOs) in high and low 2′-FL level groups from 0 to 400 days postpartum (median (p25, p75)) (mg/L).

HMOs	High 2′-FL Level Group					Low 2′-FL Level Group				
	0–5 Days (*n* = 75)	10–15 Days (*n* = 78)	40–45 Days (*n* = 84)	200–240 Days (*n* = 77)	300–400 Days (*n* = 73)	0–5 Days (*n* = 21)	10–15 Days (*n* = 18)	40–45 Days (*n* = 20)	200–240 Days (*n* = 23)	300–400 Days (*n* = 19)
2′-FL	3263 ^a^ (2553, 5109)	2446 ^b^ (1952, 2911)	2313 ^b^ (1811, 2930)	1238 ^c^ (941, 1671)	1152 ^c^ (893, 1604)	64 ^x^ (47, 86)	39 ^x^ (37, 46)	14 ^y^ (6, 18)	15 ^y^ (3, 20)	11 ^y^ (8, 13)
3-FL	198 ^c^ (135, 321)	170 ^c^ (122, 2423)	397 ^b^ (295, 587)	1148 ^a^ (823, 1550)	1037 ^a^ (781, 1296)	944 ^y^ (682, 1270)	758 ^y^ (681, 962)	1641 ^x^ (1224, 1902)	2336 ^x^ (1898, 2717)	1911 ^x^ (1533, 2395)
LNT	538 ^b^ (263, 1059)	1393 ^a^ (984, 1380)	617 ^b^ (451, 901)	287 ^c^ (193, 394)	328 ^c^ (229, 457)	2021 ^x,y^ (1164, 2366)	2299 ^x^ (1812, 2677)	1104 ^y^ (818, 1305)	443 ^z^ (316, 628)	595 ^y,z^ (374, 783)
LNnT	272 ^a^ (199, 407)	209 ^b^ (142, 1380)	142 ^c^ (87, 197)	53 ^d^ (27, 103)	56 ^d^ (24, 84)	211 ^x^ (154, 265)	115 ^x,y^ (75, 159)	49 ^y^ (30, 85)	19 ^y^ (13, 36)	19 ^y^ (11, 28)
3′-SL	246 ^a^ (207, 314)	140 ^b^ (123, 214)	112 ^c^ (96, 130)	119 ^c^ (99, 138)	136 ^b, c^ (111, 160)	216 ^x^ (197, 251)	141 ^y^ (126, 170)	111 ^y,z^ (95, 134)	103 ^z^ (90, 125)	143 ^y^ (128, 164)
6′-SL	404 ^b^ (303, 501)	611 ^a^ (521, 609)	300 ^b^ (218, 373)	36 ^c^ (22, 53)	23 ^c^ (15, 40)	431 ^x,y^ (318, 558)	568 ^x^ (529, 722)	299 ^y^ (3228, 358)	44 ^z^ (35, 59)	22 ^z^ (16, 29)
Total HMOs	5439 ^a^ (4882, 6766)	5128 ^a^ (4735, 5125)	4179 ^b^ (3707, 4541)	3095 ^c^ (2757, 3340)	2932 ^c^ (2684, 3156)	3678 ^x,y^ (3429, 4206)	3912 ^x^ (3639, 4396)	3270 ^y^ (2991, 3464)	2938 ^y^ (2759, 3398)	2817 ^y^ (2487, 3195)

2′-FL, 2′-fucosyllactose; 3-FL, 3-fucosyllactose; LNT, lacto-N-tetraose; LNnT, lacto-N-neotetraose; 3′-SL, 3′-sialyllactose; 6′-SL, 6′-sialyllactose. Total HMO concentration was calculated as the sum of 2′-FL, 3-FL, LNT, LNnT, 3′-SL, and 6′-SL. ^a,b,c,d^ values within a row in individual HMOs with unlike superscript letters were significantly different (*p* < 0.05) according to an independent nonparametric test (Kruskal–Wallis one-way ANOVA, all pairwise). ^x,y,z^ values within a row in individual HMOs with unlike superscript letters were significantly different (*p* < 0.05) according to an independent nonparametric test (Kruskal–Wallis one-way ANOVA, all pairwise).

**Table 4:** Comparison with HMO range in published literature (mg/L or mg/Kg).

	2′-FL	3-FL	LNT	LNnT	3′-SL	6′-SL	Literatures
Our study (0–400 days postpartum)	1013–2891	193–1421	314–1478	44–255	111–241	23–602	
Other Chinese study (5–240 days postpartum)	1400–2500	250–1100	190–790	49–170	75–110	42–350	[35]
Other countries’ studies (0–365 days postpartum)	710–3750	132–1588	250–2393	50–1420	80–342	39–718	[24,26,27,34,38,39]

2′-FL, 2′-fucosyllactose; 3-FL, 3-fucosyllactose; LNT, lacto-N-tetraose; LNnT, lacto-N-neotetraose; 3′-SL, 3′-sialyllactose; 6′-SL, 6′-sialyllactose.

## Data Availability

The data are available in a publicly accessible repository.
